# Differentiating depression using facial expressions in a virtual avatar communication system

**DOI:** 10.3389/fdgth.2023.1080023

**Published:** 2023-03-10

**Authors:** Ayumi Takemoto, Inese Aispuriete, Laima Niedra, Lana Franceska Dreimane

**Affiliations:** ^1^ Faculty of Computing, University of Latvia, Riga, Latvia; ^2^ Bioinformatics Laboratory, Riga Stradins University, Riga, Latvia; ^3^ Faculty of Pedagogy, Psychology and the Arts, University of Latvia, Riga, Latvia

**Keywords:** facial expression, depression detection, human-computer interaction, non-verbal information, non-clinical conversation

## Abstract

Depression has a major effect on the quality of life. Thus, identifying an effective way to detect depression is important in the field of human-machine interaction. To examine whether a combination of a virtual avatar communication system and facial expression monitoring potentially classifies people as being with or without depression, this study consists of three research aims; 1) to understand the effect of different types of interviewers such as human and virtual avatars, on people with depression symptoms, 2) to clarify the effect of neutral conversation topics on facial expressions and emotions in people with depression symptoms, and 3) to compare verbal and non-verbal information between people with or without depression. In this study, twenty-seven participants—fifteen in the control group and twelve in the depression symptoms group—were recruited. They were asked to talk to a virtual avatar and human interviewers on both neutral and negative conversation topics and to score PANAS; meanwhile, facial expressions were recorded by a web camera. Facial expressions were analyzed by both manual and automatic analyses. In the manual analysis, three annotators counted gaze directions and reacting behaviors. On the other hand, automatic facial expression detection was conducted using OpenFace. The results of PANAS suggested that there was no significance between different interviewers’ types. Furthermore, in the control group, the frequency of look-downward was larger in negative conversation topics than in neutral conversation topics. The intensity of Dimpler was larger in the control group than in the depression symptoms group. Moreover, the intensity of Chin Raiser was larger in neutral conversation topics than in negative conversation topics in the depression symptoms group. However, in the control groups, there was no significance in the types of conversation topics. In conclusion, 1) there was no significance between human and virtual avatar interviewers in emotions, facial expressions, and eye gaze patterns, 2) neutral conversation topics induced less negative emotion in both the control and depression symptoms group, and 3) different facial expressions’ patterns between people with, or without depression, were observed in the virtual avatar communication system.

## 1. Introduction

Mental or mood disorders (1) have been two of the most common health problems negatively affecting the quality of life as well as the longevity of the global population; these problems include depression, schizophrenia, anxiety disorder, and bipolar disorder (2). Depression has a significant impact on the overall quality of life not only for younger adults but also for older adults (3).

Surveys using questionnaires (4–6), or clinical interviews (7, 8) have been two of the main methods used to diagnose depression for several decades; however, current studies proposed many alternative approaches for detecting mental illnesses using social network data (9, 10), brain activity (11, 12), or behavioral patterns (13, 14). Furthermore, support systems for patients with depression using robots (15, 16), or virtual avatars (17, 18) instead of human support provision have been highlighted in recent scientific studies.

Human-machine interaction technology, such as a virtual avatar or robot communication systems, has been reported as one of the main methods of intervention for people with depression for many decades (16, 18). Pinto et al. (18) proposed a virtual avatar-based self-management intervention system—eSMART-MH, which provides a virtual health coach to practice communication for patients with depression. These patients reported a reduction in depressive symptoms after using eSMART-MH for three months (18). Hung et al. (16) reviewed the effectiveness of social robots in mental care, reporting that they reduce negative emotion, improve social engagement, and promote positive moods in patients with depression.

When classifying people as being with, or without depression, not only verbal but also non-verbal information collected by cameras, such as facial expressions, eye movements, and behavioral patterns have been increasingly paid more attention in recent decades. It was reported that speech styles such as words or pause duration are one of the criteria required to identify people with depression symptoms (19, 20). Cummins et al. (20) summarized that reduced pitch/pitch-range/speaking-intensity/intonation, slower speaking ratio, and lack of linguistic stress represented the depressed-speech style. On the other hand, Islam et al. (9) reported that social network data analysis by machine learning technology is one of the most effective approaches to detecting people with depression highlighting three main factors that had an impact on detecting depression through social network data: linguistic style (e.g. adverbs, conjunctions, pronouns, verbs), emotional process (e.g. positive, negative, sad, anger, anxiety), and temporal process (present focus, past focus, and future focus). The accuracy of a machine learning model using each factor is greater than 60%, thus, comments posted on social networks have been established as one of the criteria used to detect depression (9). Furthermore, non-verbal information, especially facial expressions, can be effectively used to detect depression (3, 21–24). People with depression display fewer positive facial expressions, such as smiling, and more negative facial expressions than people without depression whilst watching positive films, however, during negative films, people with depression make fewer negative facial expressions than people without depression (24). Girard et al. (21) developed a system to detect the severity of depression symptoms using a Facial Action Coding System (FACS) during a series of clinical interviews which confirmed that patients with high-severity symptoms of depression exhibit more negative facial expressions, such as contempt, and smiled less.

Facial expressions or gaze patterns have been employed to understand brain activity or to detect mental conditions; on the other hand, the effects of different interviewers, or different conversation topics on gaze patterns, facial expressions, emotions, or other non-verbal information remain unclear.

Several studies have reported the effect of the robot or virtual avatar interviewer on human emotion, empathy, or action (25–29). People perceived the robot which exhibited a more empathetic attitude and verbal utterance, as friendlier (27). In addition, Appel et al. (25) reported that congruence between robots’ facial expressions and verbal information would lead to a more positive impression on users. A virtual avatar interviewer invokes stress as much as a human interviewer in the various tasks (28). However, the effect of computer interaction on human emotion or action is still not clear in people with depression symptoms. Thus, the first research aim of this study is to understand the different effects that human and virtual avatar interviewers have on facial expressions and emotions in people with depression.

In past studies, clinical interviews/tasks and negative conversation topics such as depression or military experiences were primarily used for studies focusing on detecting mood, or mental disorders, such as depression, post-traumatic stress disorder (PTSD), or attention deficit hyperactivity disorder (ADHD) (30–34). On the other hand, clinical interviews, including disclosure of feelings and problem-solving, induced more anxiety, depression, and behavioral fear than unrelated conversation topics (35), and it is also still unclear whether neutral conversation topics are effective in differentiating people with or without depression symptoms. Thus, the second aim was to clarify the effect of neutral conversation topics (e.g. non- clinical, military, and depression interviews) on facial expressions and emotions in people with depression.

Past studies have reported the effect of virtual avatars on people’s emotions, rapport, or decision-making (36–38). Furthermore, eye gaze patterns and facial expressions are two of the main criteria used to identify people’s emotions. Chen et al. (39) highlighted that a downward gaze was perceived as a negative social signal and enhanced the startled response magnitude. In facial expressions, it was reported that negative emotions, such as disgust, fear, boredom, or sadness reflected the intensity of the Lip Corner Depressor and Brow Lowerer (40–43), and positive emotions, such as joy, reflected the intensity of Cheek Raiser and Lip Corner Puller (40). However, communication systems for people with depression have been still developing, and it is still not clear whether non-verbal information, such as eye gaze patterns or facial expressions while interacting with the virtual avatar, is effective in differentiating people with depression symptoms. The final aim of this study was to compare verbal and non-verbal information between people with, and without depression symptoms while talking to the virtual avatar about non-clinical interview topics.

In this experiment, twenty-seven participants (fifteen in the control group and twelve in the depression symptoms group) were asked to talk to each human, or virtual avatar interviewer on each, negative or neutral, conversation topic through a monitor; meanwhile, eye movements, heart rate, facial expression, verbal, and non-verbal information were recorded.

## 2. Materials and methods

The pre-registration of this research has been registered in Open Science Framework (OSF) (Registration DOI:10.17605/OSF.IO/B9DNE). These experiments were conducted with participants interacting with two types of interviewers through a monitor on two types of conversation topics. Participants performed conversation tasks with each interviewer. This study was approved by the Ethics Committee of the University of Latvia in accordance with the Declaration of Helsinki (approval number: 30-47/18). This research has focused on the effect of the types of ***interviewers*** (human or virtual avatar interviewer) and ***conversation topics*** (neutral, or negative topics) on facial expressions, eye gaze patterns, and verbal information in both ***types of participants*** (people with or without depression), thus this paper has analyzed the results of the collected facial expressions and verbal data by a web-camera.

### 2.1. Surveys

#### 2.1.1. Positive and Negative Affect Schedule

The Positive and Negative Affect Schedule (PANAS) consists of twenty-item scales to measure both positive and negative effects (44), and each item can be rated from 1 (not at all) to 5 (very much). The reliability of this survey to measure the emotional effect was reported in many different types of medical situations such as rehabilitation or clinical interviews (45, 46). Furthermore, the psychometric properties of the scale were clarified in clinical samples with anxiety, depressive, and adjustment disorders in recent years (46). The effects of each experimental condition on participants’ emotions were assessed by asking the Latvian version of PANAS (47) before and after each session.

#### 2.1.2. Patient Health Questionnaire-9

A patient Health Questionnaire-9 (PHQ-9) consists of nine items which can be scored from 0 (not at all) to 3 (nearly every day), to screen for depression. Kroenke et al. (4) reported that PHQ-9 score ≥10 has a specificity of 88% for major depression. Furthermore, cut-off scores between 8 and 11 have no significance in sensitivity and specificity (48). In this study, a score of 10 as the most common cut-off score, was used as a cut-off score. Participants filled in a PHQ-9 in Latvian (49) before starting the experiment.

#### 2.1.3. International Personality Item Pool – Five Factor Model – 50 (IPIP-Big5)

International Personality Item Pool – Five Factor Model – 50 (IPIP-Big5) (50, 51) is used to classify and compare personality traits in many types of languages (52, 53). Furthermore, past studies reported that IPIP-Big5 was studied in people with depression (54, 55). The IPIP-Big5 translated and verified by Pērkona and Koļesovs (56) based on Perepjolkina and Reņǵe (57) and Schmitt et al. (58) was used in this experiment. The questionnaire consists of a fifty-item scale, and each item can be scored from 1 (Disagree strongly) to 5 (Agree strongly). The five basic dimensions of personality were based on the study published by Strus et al. (51).

### 2.2. Participants

All participants were native Latvian speakers and were recruited and screened using PHQ-9 through Social Networking Service (SNS). A priori power analysis (G*Power ver 3.1 59) was conducted to determine the small sample size, and this indicated that the required sample size was a mere twelve people for each control group and depression symptoms group. Participants answered PHQ-9 on their experimental dates again and were classified as being in the control group (the score of PHQ-9 is lower than 10) or the depression symptoms group (the score of PHQ-9 is 10 or higher). Overall, participants for both the control group (N=17) and the depression symptoms group (N=13) took part in the experiment. Each participant provided written informed consent before the experiment and received a gift worth approximately 12 USD. A male participant in the depression symptoms group, whose PHQ-9 answered through SNS was higher than the cut-off score and was found to have it lower on the day that he participated in the experiment, and female and male participants in the control group, who experienced a technical issue in the middle of experiments, were excluded from all of the analysis.

### 2.3. Apparatus

The interviewers were presented on a monitor (Lenovo, 2880×1620 pixels, 34.31×19.30 cm) and controlled by a native Latvian member of the experiment team through a Unity game engine in the same room. The viewing distance was 60 cm. Eye movements were recorded by Tobii Pro Nano with a 60 Hz refresh rate and calibrated before each session by the Tobii Pro Lab. Facial expressions were monitored by the web-camera (Logitech—the C270 HD Webcam, 720p/30fps), and body language was recorded by the RGB camera (Canon EOS 1100 D, 25 fps). In addition, heart-beat ratios were monitored using a smartwatch (Fitbit Versa 2) every five seconds, but the research has focused on facial expressions data in this paper, and thus, the data of heart-beat ratios, eye tracking, and body language were not used.

### 2.4. Experimental setup

**Figure 1:**
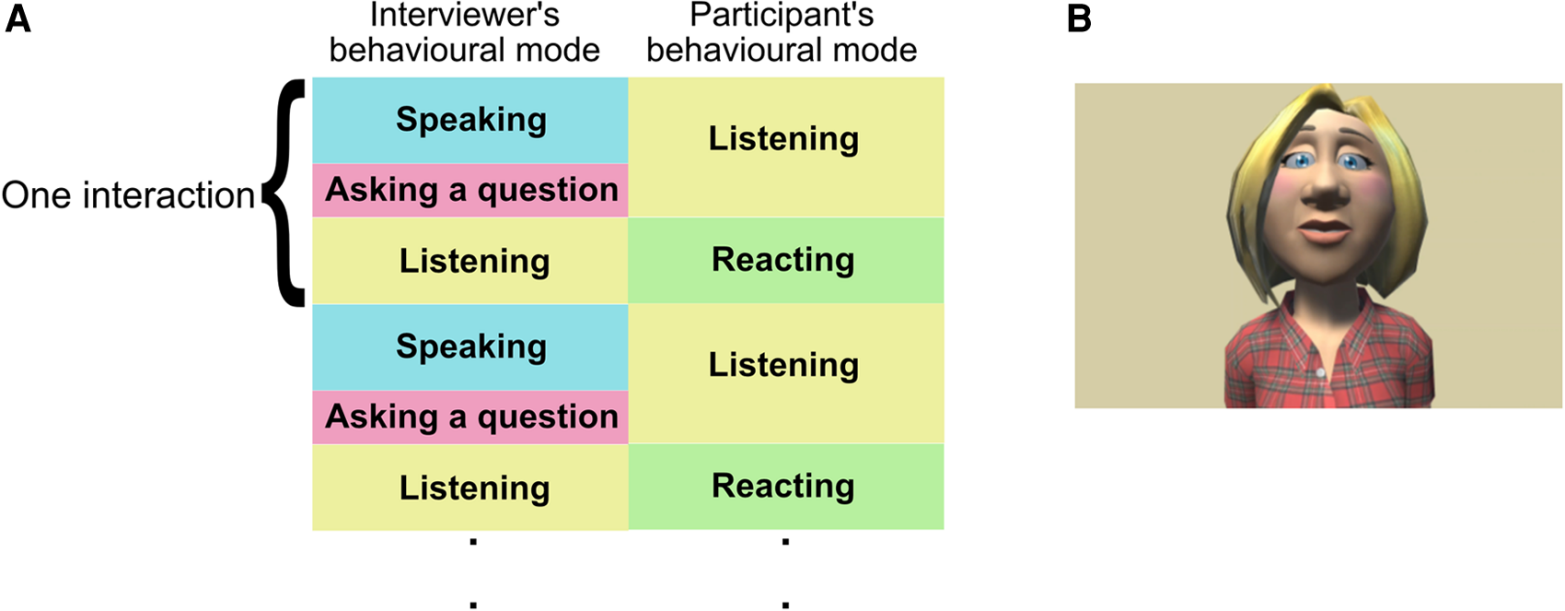
(**A**) Experimental flow and behavior modes of participants and interviewers and (**B**) the appearance of the virtual avatar interviewer.

Interaction of the conversation task involved roughly structured dialogues between the participant and the interviewer (Figure 1A). Each session consisted of thirty trials and; each trial had two modes based on participants’ behavior—a listening mode where the interviewer led the conversation with a closed-end question (participants can answer “yes” or “no”) based on the topic and a reacting mode where the participant was asked to answer the question in five seconds (Figure 1A). Two members of the research team were in the same room as the participants and controlled the experimental system based on the participants’ reactions. Participants were offered a break between sessions. In the case of the human interviewer, if participants took over ten seconds to answer the questions, the video which was playing was automatically stopped until the system was moved to the next trial by a member of the research team. Each participant performed a total of four sessions—two types of conversation topics (neutral and negative) with two types of interviewers (human and virtual avatar). The order of the combination of the ***types of conversation topics*** and the ***interviewers*** was assigned randomly to participants. Before starting the main session, participants had practiced talking to the virtual avatar interviewer about animals in five trials. In order to clarify the effect of each experimental condition, participants were asked to fill in a PANAS before and after each session.

### 2.5. Interviewers

Two types of interviewers were prepared; an animated type of virtual avatar and a human to clarify the differences between them. Several recent studies already reported that there is no significance in the type of virtual avatar (e.g. a human-like and animated avatar), age, gender, and ethnicity of virtual avatars in frustration levels, preference, and the level of rapport (60–62). For the determination of a virtual avatar, several different types of animated virtual avatars’ pictures that resembled the general Latvian appearance (e.g. light skin tone, blue eyes, and blonde hair), current casual clothing, and hairstyle were developed using Toon people ver 3.1 which is a Unity asset produced by JBGarraza (63). Students in the Department of Psychology scored their impression using the 5-point Likert scale (61), and the virtual avatar which had the most positive impression on participants, was used in the interacting experiment (Figure 1B). The voice data of the virtual avatar interviewer were produced by software (64) and Hugo.lv which was used as the online text-to-speech application (65) to convert the written text into spoken words for the Latvian language. The virtual avatar interviewer was computed to blink four or three times per ten seconds based on the average natural human blinking ratio (66,67) and to move the mouth based on sentences.

In the listening mode, interviewers talked to participants and asked questions, and participants listened to this. In the reacting mode, interviewers nodded, and participants answered the questions. In the case of the human interviewer, the videos were prepared in that a native Latvian had spoken the same sentence as the virtual avatar and afterward the human interviewer nodded for approximately ten seconds which was twice as long as the length the participants were asked to talk. They, then, were played in order, and participants interacted with the video through a monitor.

### 2.6. Conversation strategies

Two types of conversation topics were prepared in Latvian—negative topics (war and loneliness) as it has been reported that these topics have a high impact on vocal, visual, and verbal features used to detect depression (32) and neutral topics (gardening and traveling).

### 2.7. Verbal and non-verbal behavior annotation

Several non-verbal behaviors of participants were annotated, such as gaze direction (up, down, side, and rotation) and reacting behaviors (smile, nodding, and shaking head) while participants were answering the interviewers’ questions. Table 1 indicates the criteria for annotating. Moreover, the number of words that participants used when talking to the interviewers, was counted. Four students of the University of Latvia were hired as annotators, and all data were annotated by three annotators using ELAN (68, 69). The average data were computed using three annotators’ data. In the human interviewer’s case, the number of words before stopping videos was used for analysis because of the possible effect of stopping the video on words’ frequency.

**Table 1:** Annotation.

Category	Features
Look-up	Participants looked upper-side
Look-downward	Participants looked lower-side
Look-averted	Participants looked horizontal-side. No matter which sides; left or right
Look-rotating	Participants rotated their eyes at least 180-degree
Nodding	The head was tilted downward or forward
Smile	The corners of mouth curved slightly upward
Shaking head	Participants turned their head from side to side

### 2.8. Facial expression analysis using OpenFace

**Figure 2:**
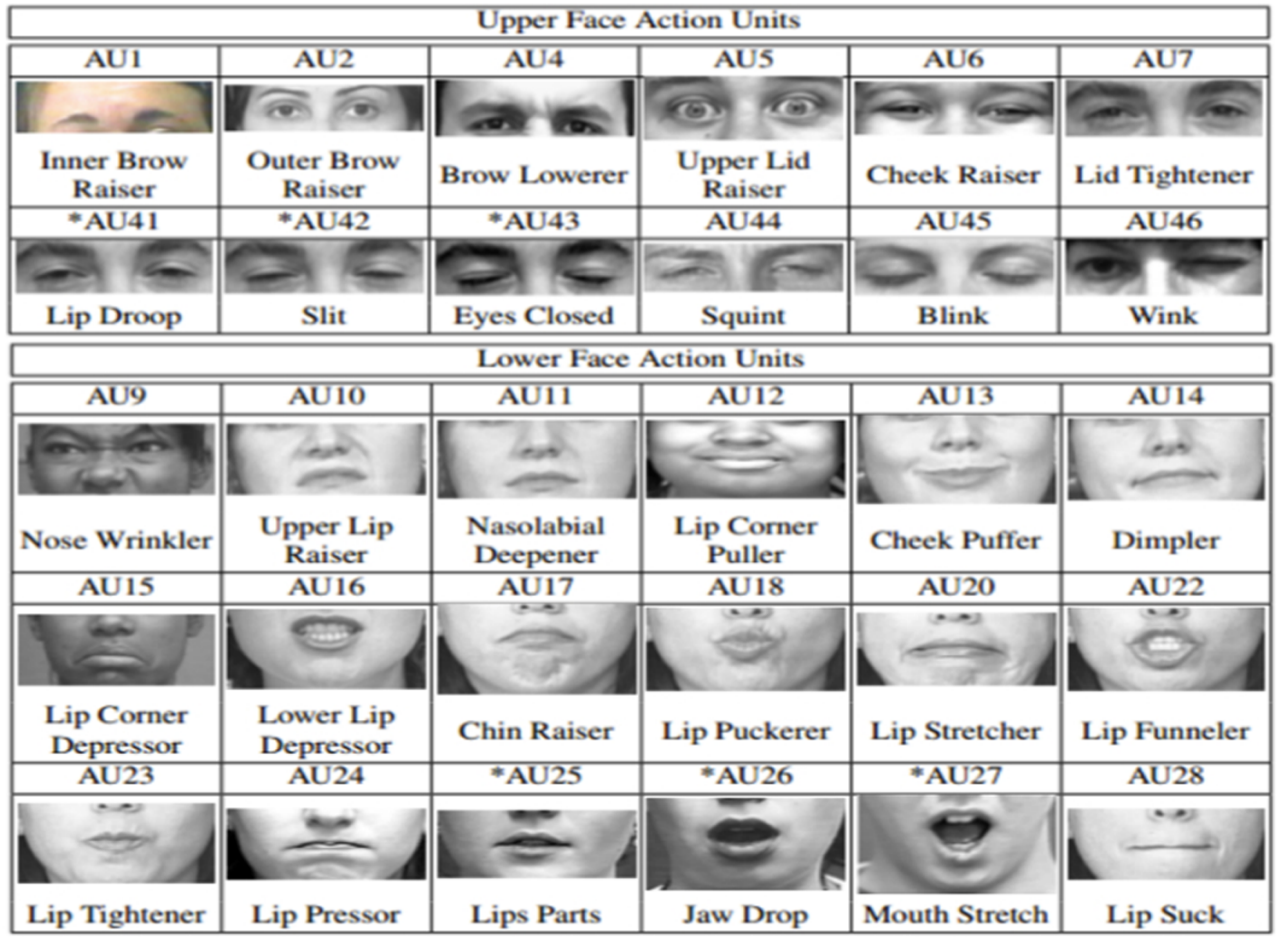
The criteria of facial action units (73, 74).

Facial landmarks, head poses, facial action units (Figure 2), and gaze directions were detected by OpenFace toolkit for emotion recognition using videos of the events that cause reaction (70–72).

### 2.9. Statistical analysis

Three-way analysis of variance (ANOVA) within/between interactions was conducted with ***types of participants***, ***interviewers***, and ***conversation topics*** as the main factors. In the ANOVAs of this study, a Huynh-Feldt correction was applied when the assumption of sphericity was not met by the Mendoza test. 95% confidential interval (CI) was computed based on Loftus and Masson’s procedure.

## 3. Results

A posthoc analysis was conducted by G*Power (59) to confirm sufficient statistical power (Power = .945). Tables 2 and 3 indicate the socio-demographics data in each participant’s group. This section reports the results of the effect of experimental conditions on participants’ mood changes before and after and facial expression differences analyzed by both manual and automatic methods by examining ***types of participants*** (control vs depression symptoms group), ***interviewers*** (human vs virtual avatar), and ***conversation topics*** (neutral vs negative).

**Table 2:** Participants’ traits.

	Control group	Depression symptoms group
Total number of participants	15	12
Female number	8	9
Age range	20–48 (30.75±8.90)	20–47 (28.25±8.69)
PHQ-9 score range	2–9 (5.47±1.92)	10–23 (14.08±4.38)

**Table 3:** Big 5 results in each group.

	Control group	Depression symptoms group	P (T<=t) two -tail
Extraversion	M=3.04, S.D.=0.59	M=2.34, S.D.=0.89	p=.057
Agreeableness	M=3.62, S.D.=0.81	M=3.49, S.D.=0.75	p=.711
Conscientiousness	M=3.52, S.D.=0.55	M=3.15, S.D.=1.04	p=.285
Emotional stability	M=3.33, S.D.=0.39	M=2.32, S.D.=0.71	p=.001∗
Openness to experience	M=3.63, S.D.=0.19	M=3.35, S.D.=0.64	p=.261

*p < 0.002.

### 3.1. Comparison Positive and Negative Affect Schedule (PANAS) between participants and experimental conditions in each type of participant’s group

**Figure 3:**
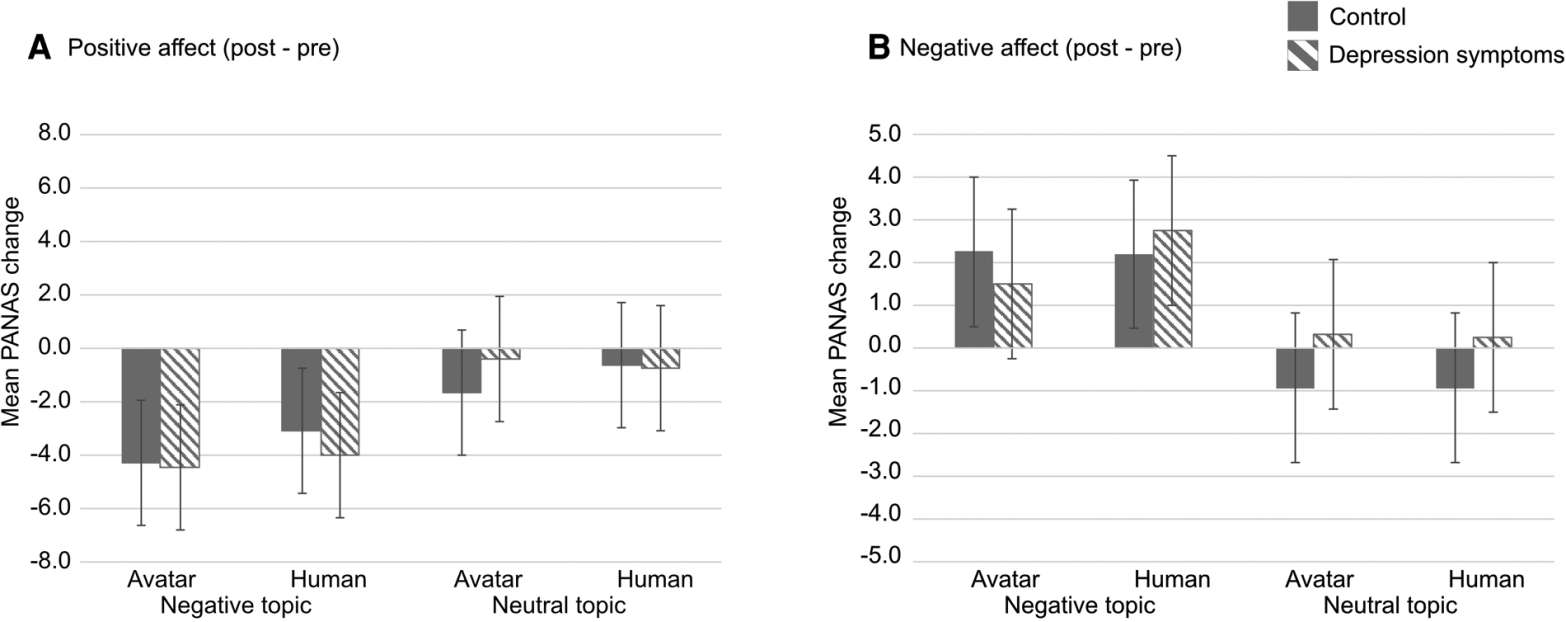
Result indicates the change of PANAS’s score between post- and pre-session in PANAS. (**A**) positive affect and (**B**) negative affect. Striped pattern boxes indicate the data of the depressed symptoms group, and solid boxes are the control group. Error bars indicate 95% CI.

The results of changes in PANAS scores which measured the effect of ***types of conversation topics*** and ***interviewers***, between before (pre) and after (post) each session indicated that there was no significant interaction between ***types of participants***, ***interviewers***, and ***conversation topics*** in both the score of positive (Figure 3A) and negative effects (Figure 3B). However, the main effect of ***types of conversation topics*** were significant in the change of both positive and negative affect’s score (F(1,25)=8.1792, p=.0084, $\eta^{2}=.1183$, F(1,25)=11.2158, p=.0026, $\eta^{2}=.1356$, respectively). The change of positive affect’s score was much lower in negative rather than neutral conversation topics, and that of the negative affect’s score was higher in negative rather than neutral conversation topics. The difference of ***types of conversation topics*** had a large impact on both the control and depression symptoms group.

### 3.2. Comparison annotation results in each participants’ group type

#### 3.2.1. The frequency of look-downward and look-averted

**Figure 4:**
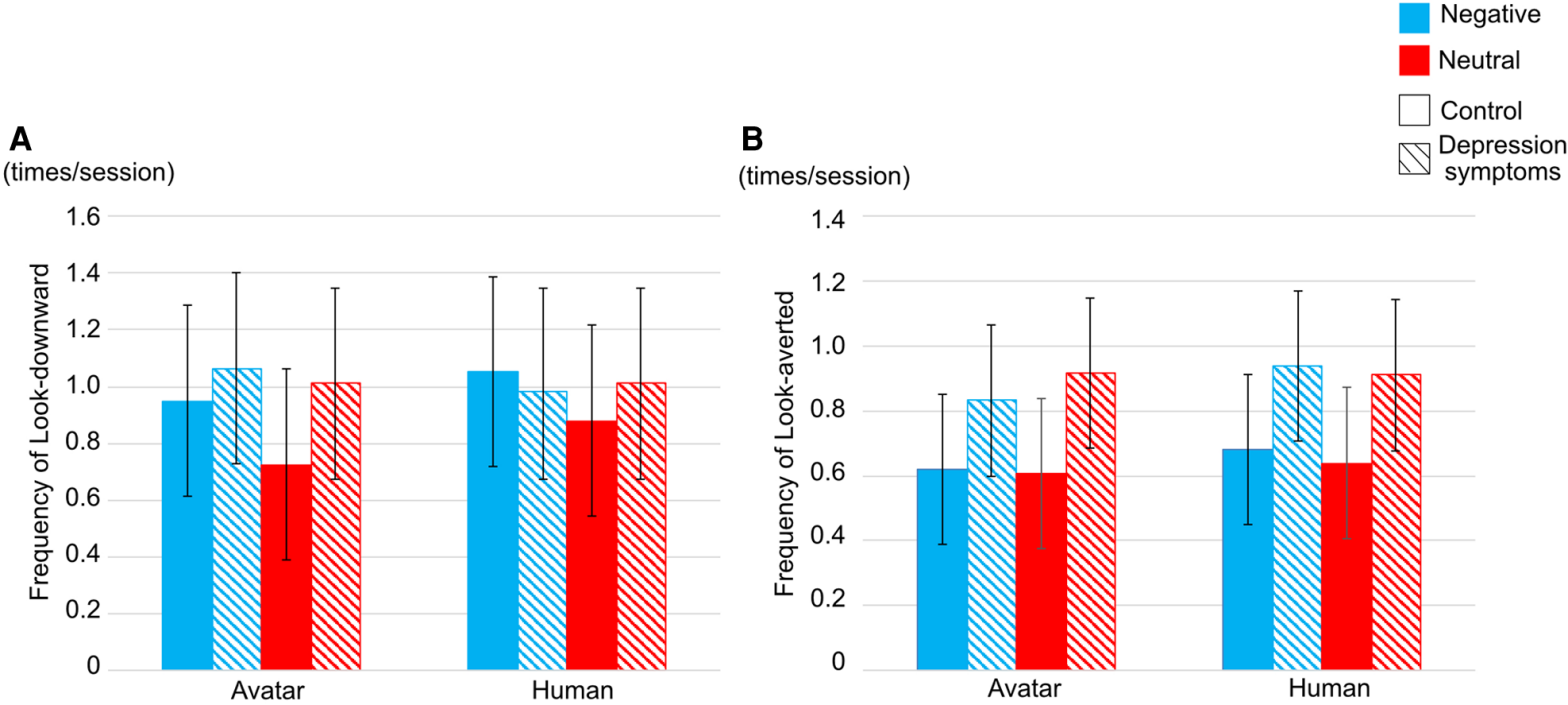
Frequency of (**A**) look-downward and (**B**) look-averted in each interviewer. Striped pattern boxes indicate the data of the depressed symptoms group, and solid boxes are the control group. Error bars indicate 95% CI.

With regard to the frequency of look-downward, there was no interaction among the three factors; however, there was significance in the main effects of ***types of conversation topics*** (F(1,25)=4.6196, p=.0415, $\eta^{2}=.0075$). In ***types of conversation topics***, the frequency of look-downward was lower in the neutral conversation topics than in the negative conversation topics (Neutral topics: Mean (M)=0.8949, Standard deviation (S.D.)=0.5903; Negative topics: M=1.0109, S.D.=0.6134) (Figure 4A).

In the frequency of look-averted, there was no interaction between ***types of participants***, ***interviewers***, and ***conversation topics*** and the main effect of each factor (Figure 4B).

#### 3.2.2. The frequency of words

**Figure 5:**
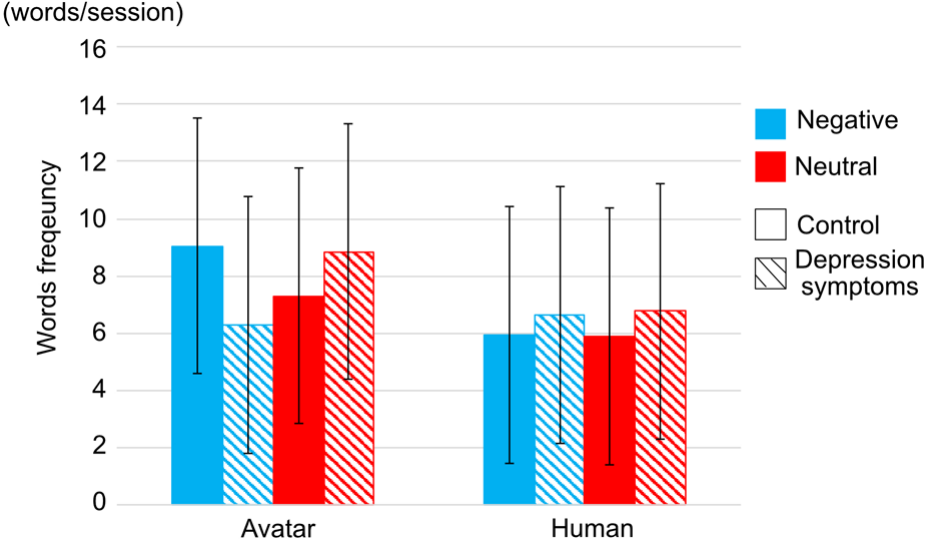
Word frequency in each interviewer. Striped pattern boxes indicate the data of the depressed symptoms group, and solid boxes are the control group. Error bars indicate 95% CI.

The frequency of words to which participants responded in reacting duration was reported (Figure 5). There was no interrelation between ***types of participants***, ***types of interviewers***, and ***types of conversation topics***, however, there is significant interrelation between ***types of participants*** and ***conversation topics*** (F(1,25)=4.2526, p=.0497, $\eta^{2}=.0049$).

### 3.3. Comparison of Facial Action Coding System (FACS) data in each participants’ group type

The intensity (from 0 to 5) of seventeen Action Units (AUs), the gaze angle of the averted and vertical axis, and the differences in head rotations by Open-Face were all computed. In the results, there was significance in the intensity of Dimpler (AU 14), Lip Corner Depressor (AU 15), and Chin Raiser (AU 17) in ***types of participants***.

**Figure 6:**
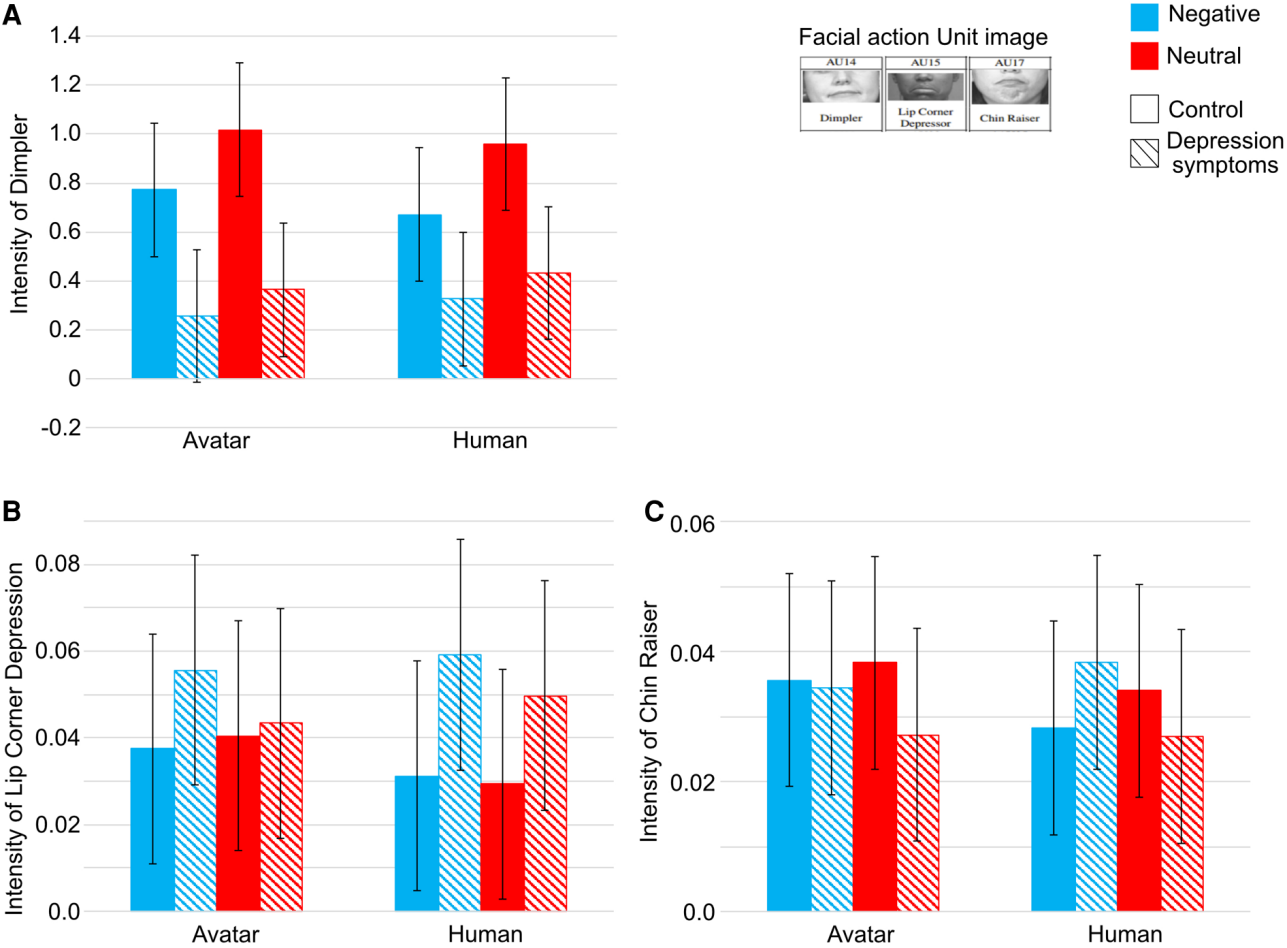
Intensity of (**A**) Dimpler (**B**) Lip Corner Depressor, and (**C**) Chin Raiser. Striped pattern boxes indicate the data of the depressed symptoms group, and solid boxes are the control group. Error bars indicate 95% CI.

Figure 6A indicates the intensity of Dimpler. There were interactions between ***types of participants*** and ***interviewers*** (F(1,25)=4.5909, p=.0421, $\eta^{2}=.0045$). In the interaction between ***types of participants*** and ***interviewers***, there was no significance between ***types of interviewers*** in each participants’ group, however, the intensity of Dimpler was higher in the control group than in the depression symptoms groups in both ***types of interviewers*** (avatar: F(1,25)=9.4350, p=.0051, $\eta^{2}=.2540$; human: F(1,25)=5.5984, p=.0260, $\eta^{2}=.1660$). Furthermore, there was significance in the main effects of ***types of participants*** and ***conversation topics***. The intensity of Dimpler was higher in the control group than in the depression symptoms group (Control group: M=0.8554, S.D.=0.5921; Depression symptoms group: M=0.3448, S.D.=0.6134; F(1,25)=7.6888, p=.0103, $\eta^{2}=.2092$), and it was higher in neutral conversation topic than negative conversation topics (Neutral topics: M=0.7261, S.D.=0.5991; Negative topics: M=0.5308, S.D.=0.4894; F(1,25)=23.0747, p=.0001, $\eta^{2}=.0279$).

Next, with regard to the intensity of the Lip Corner Depressor, there was no significance between ***conversation topics***, ***types of participants*** and ***interviewers*** and the main effect in each factor (Figure 6B).

Finally, with regard to the intensity of the Chin Raiser, there was significance between ***types of participants*** and ***conversation topics*** (F(1,25)=6.5944, p=.0166, $\eta^{2}=.0130$) (Figure 6C). In the depression symptoms group, the intensity of Chin Raiser was lower in neutral conversation topics than in negative conversation topics (F(1,11)=9.6061, p=.0101, $\eta^{2}=.0320$).

## 4. Discussion

In this section, the effects of ***types of interviewers*** and ***conversation topics*** on verbal, facial expression, and annotated gaze patterns in ***types of participants***, such as control and depression symptoms groups, are interpreted based on the three aims presented in the Introduction section: (1) to understand the different effects of ***types of interviewers***, (2) to clarify the effect of neutral conversation topics on facial expressions and emotions in people with depression, and (3) to compare verbal and non-verbal information between people with, or without depression.

### 4.1. Understanding of the effect of the different types of interviewers

With regard to the effect of the different ***types of interviewers***, the virtual avatar had no impact on emotions, facial expressions, and eye gaze directions on people with or without depression. With regard to the PANAS score, there was no significance between the different ***types of interviewers***, and the result is consistent with what Schneeberger et al. (28) suggested. They reported that female participants followed the virtual avatar at the same level as the human interviewer and completed the various tasks. Both the virtual avatar and the human interviewers invoke feelings of stress to the same level. The PANAS of these results indicated that the virtual avatar has the same level of emotional impact as the human interviewer in both ***types of conversation topics***. It is, therefore, suggested the virtual agent has the same authority as the human interviewer; thus, the virtual avatar would be effective in the interview process with depressed patients. However, an animated virtual avatar as a virtual avatar interviewer and a recorded video as a human interviewer were used in this study, thus, the emotional effect of a human-like virtual avatar and real-human interviewers are still unclear on people with depression.

### 4.2. Clarifying the effect of the different types of conversation topics

The effect of the different ***types of conversation topics*** was shown in the PANAS score, the frequency of look-downward and words, and the intensity of Dimpler and Chin Raiser.

First, the PANAS score indicated the emotional effect of the different ***types of conversation topics*** on participants in both the control and the depression symptoms groups. In negative conversation topics, the post-scores of positive effects were much lower after the experiment than the pre-scores in both the control and depression symptoms group and vice versa. The results are consistent with the suggestions of Costanza et al. (35). Positive conversation topics led to fewer negative emotions than negative conversation topics in both ***types of interviewers*** and ***types of participants***.

Secondly, the frequency of look-downward was higher in negative conversation topics than in neutral conversation topics in both ***types of participants***. Gaze patterns are primarily social cues used to represent emotions. Look-downward shows more negative social signals than direct gaze in psychophysiology (39). The result of gaze patterns in ***types of conversation topics*** in this study is consistent with these past studies. It was concluded that negative conversation topics induced participants’ negative emotions, thus participants tended to look downward more frequently than in neutral conversation topics.

The intensity of Dimpler was lower, and that of Chin Raiser was higher, in negative conversation topics than neutral conversation topics in both control and depression symptoms groups. The intensity of Dimpler, Lip Corner Depressor, and Chin Raiser generally represent emotional expression, especially as the intensity of Dimpler increases and that of Lip Corner Depressor and Chin Raise decreases when people have negative emotions such as disgust, anger, sadness, or fear (40, 42). The results of PANAS and the intensity of Dimpler and Chin Raiser in this study were consistent with these past studies. ***Types of conversation topics*** had a large effect on both the control and depression symptoms groups, namely negative conversation topics induced negative emotion in both types of participants. Thus, it was concluded that negative emotions induced the lower intensity of Dimpler and the higher intensity of Chin Raiser. The limitation of this study is that a closed-end question (participants can only answer “yes” or “no”) was used to restrict the answers of participants in controlling the experimental duration, thus it is unclear whether an open-ended question that cannot be answered with “yes” or “no” has any effect on non-verbal information or emotions in people with depression.

### 4.3. Comparison of verbal and non-verbal information between people with and without depression

In ***types of participants***, the frequency of words and the intensity of Dimpler and Chin Raiser have differences between control and depression symptoms groups.

First, the frequency of words was larger in neutral conversation topics than in negative conversation topics in the depression symptoms group. Several past studies reported that depression was characterized by speech style, especially people with depression who tend to have fewer social interactions and have difficulty choosing words (19,20). However, to the best of our knowledge, no past studies reported the behavioral pattern of people with depression in neutral conversation topics that are not clinically related conversation topics. It is, therefore, concluded that negative conversation topics were difficult for people with depression to choose words in answering the questions, thus, people with depression symptoms talked less than people in the control group in negative conversation topics.

Secondly, the intensity of Dimpler was higher in the control group than in the depression symptoms group. The intensity of Dimpler, Chin Raiser, and Lip Corner Depressor were the main criteria for negative facial expressions (40, 42). Several past studies reported that the intensity of Dimpler is higher in the low severity of depression symptoms group than in high severity (22); however, other studies found opposing results. Hsu et al. (23), Rottenberg et al. (75) interpreted that depression is marked by reductions in facial expressions. Furthermore, past studies reported that the intensity of Dimpler increases when healthy people feel boredom (41, 43). It is interpreted, therefore, that the participants in the control group felt boredom more than those in the depression symptoms group, thus, it is higher in the control group. Furthermore, in the Chin Raiser, the intensity is higher in the depression symptoms group when they were talking about negative conversation topics. It was supposed that participants in the depression symptoms group had negative emotional impacts from negative conversation topics more than in the control group, thus the intensity of Chin Raiser was higher. Another limitation is that classification experiments using computer science methodologies, such as machine learning or AI were not conducted in this study.

In conclusion, based on the results of the manual (annotation) and automatic (OpenFace) non-verbal analysis, there was no significance in the different ***types of interviewers***, and people with depression symptoms would make more negative facial expressions such as Chin Raiser when they were interacting with virtual avatar interviewers with neutral conversation topics. In further studies, classification experiments using computer science methodologies would be required in order to clarify whether people with depression could be differentiated using facial expressions in virtual avatar communication with neutral conversation topics.

## Data Availability

The datasets from the current study are available from the corresponding author on reasonable request.

## References

[1] American Psychiatric Association. Diagnostic, statistical manual of mental disorders. Am Psychiatric Assoc. 5th edn: DSM-5 (2013) 21:591–643. 10.1176/appi.books.9780890425596

[2] James SL Abate D Abate KH Abay SM Abbafati C Abbasi N, et al. Global, regional,, national incidence, prevalence,, years lived with disability for 354 diseases and injuries for 195 countries and territories, 1990–2017: a systematic analysis for the global burden of disease study 2017. Lancet. (2018) 392:1789–858. 10.1016/S0140-6736(18)32279-7 30496104 PMC6227754

[3] Lawhorne L. Depression in the older adult. Prim Care. (2005) 32:777–92. 10.1016/j.pop.2005.06.001 16140127

[4] Kroenke K Spitzer RL Williams JB. The PHQ-9: validity of a brief depression severity measure. J Gen Intern Med. (2001) 16:606–13. 10.1046/j.1525-1497.2001.016009606.x 11556941 PMC1495268

[5] Norton S Cosco T Doyle F Done J Sacker A. The hospital anxiety and depression scale: a meta confirmatory factor analysis. J Psychosom Res. (2013) 74:74–81. 10.1016/j.jpsychores.2012.10.010 23272992

[6] Smarr KL Keefer AL. Measures of depression and depressive symptoms: beck depression inventory-II (BDI-II), center for epidemiologic studies depression scale (CES-D), geriatric depression scale (GDS), hospital anxiety and depression scale (HADS), and patient health questionnaire-9 (PHQ-9). Arthritis Care Res. (2011) 63:S454–66. 10.1002/acr.20556 22588766

[7] Dibeklioğlu H Hammal Z Yang Y Cohn JF. Multimodal detection of depression in clinical interviews. In *Proceedings of the 2015 ACM on International Conference on Multimodal Interaction*. Seattle: Association for Computing Machinery (ACM) (2015). p. 307–10. 10.1145/2818346.2820776 PMC4874497 27213186

[8] Stuart AL Pasco JA Jacka FN Brennan SL Berk M Williams LJ. Comparison of self-report and structured clinical interview in the identification of depression. Compr Psychiatry. (2014) 55:866–9. 10.1016/j.comppsych.2013.12.019 24467941

[9] Islam M Kabir MA Ahmed A Kamal ARM Wang H Ulhaq A, et al. Depression detection from social network data using machine learning techniques. Health Inf Sci Syst. (2018) 6:1–12. 10.1007/s13755-018-0046-0 30186594 PMC6111060

[10] Orabi AH Buddhitha P Orabi MH Inkpen D. Deep learning for depression detection of twitter users. In: *Proceedings of the Fifth Workshop on Computational Linguistics and Clinical Psychology: From Keyboard to Clinic*. Louisiana: Association for Computational Linguistics (2018). p. 88–97.

[11] Ay B Yildirim O Talo M Baloglu UB Aydin G Puthankattil SD, et al. Automated depression detection using deep representation and sequence learning with EEG signals. J Med Syst. (2019) 43:1–12. 10.1007/s10916-019-1345-y 31139932

[12] Loh HW Ooi CP Aydemir E Tuncer T Dogan S Acharya UR. Decision support system for major depression detection using spectrogram and convolution neural network with eeg signals. Expert Syst. (2022) 39:e12773. 10.1111/exsy.12773

[13] Song S Shen L Valstar M. Human behaviour-based automatic depression analysis using hand-crafted statistics and deep learned spectral features. In: *2018 13th IEEE International Conference on Automatic Face & Gesture Recognition (FG 2018)*. IEEE (2018). p. 158–165.

[14] Wang T Li C Wu C Zhao C Sun J Peng H, et al. A gait assessment framework for depression detection using Kinect sensors. IEEE Sens J. (2020) 21:3260–70. 10.1109/JSEN.2020.3022374

[15] Chen S-C Moyle W Jones C Petsky H. A social robot intervention on depression, loneliness, and quality of life for Taiwanese older adults in long-term care. Int Psychogeriatr. (2020) 32:981–91. 10.1017/S1041610220000459 32284080

[16] Hung L Liu C Woldum E Au-Yeung A Berndt A Wallsworth C, et al. The benefits of and barriers to using a social robot PARO in care settings: a scoping review. BMC Geriatr. (2019) 19:1–10. 10.1186/s12877-019-1244-6 31443636 PMC6708202

[17] Pagliari C Burton C McKINSTRY B Szentatotai A David D Ferrini L, et al. Psychosocial implications of avatar use in supporting therapy for depression. Annu Rev Cyber Telemedicine. (2012) 2012 329–33. 10.3233/978-1-61499-121-2-329 22954882

[18] Pinto MD Hickman Jr RL Clochesy J Buchner M. Avatar-based depression self-management technology: promising approach to improve depressive symptoms among young adults. Appl Nurs Res. (2013) 26:45–8. 10.1016/j.apnr.2012.08.003 23265918 PMC3551988

[19] Alghowinem S Goecke R Wagner M Epps J Hyett M Parker G, et al. Multimodal depression detection: fusion analysis of paralinguistic, head pose, eye gaze behaviors. IEEE Trans Affect Comput. (2016) 9:478–90. 10.1109/TAFFC.2016.2634527

[20] Cummins N Scherer S Krajewski J Schnieder S Epps J Quatieri TF. A review of depression, suicide risk assessment using speech analysis. Speech Commun. (2015) 71:10–49. 10.1016/j.specom.2015.03.004

[21] Girard JM Cohn JF Mahoor MH Mavadati S Rosenwald DP. Social risk and depression: evidence from manual and automatic facial expression analysis. In: *2013 10th IEEE International Conference and Workshops on Automatic Face and Gesture Recognition (FG)*. IEEE (2013). p. 1–8. 10.1109/FG.2013.6553748 PMC3935843 24598859

[22] Girard JM Cohn JF Mahoor MH Mavadati SM Hammal Z Rosenwald DP. Nonverbal social withdrawal in depression: evidence from manual and automatic analyses. Image Vis Comput. (2014) 32:641–7. 10.1016/j.imavis.2013.12.007 25378765 PMC4217695

[23] Hsu C-W Chang C-C Lin C-J. A practical guide to support vector classification (2003).

[24] Renneberg B Heyn K Gebhard R Bachmann S. Facial expression of emotions in borderline personality disorder, depression. J Behav Ther Exp Psychiatry. (2005) 36:183–96. 10.1016/j.jbtep.2005.05.002 15950175

[25] Appel M Lugrin B Kühle M Heindl C. The emotional robotic storyteller: on the influence of affect congruency on narrative transportation, robot perception, and persuasion. Comput Human Behav. (2021) 120:106749. 10.1016/j.chb.2021.106749

[26] Gockley R Simmons R Forlizzi J. Modeling affect in socially interactive robots. In: *ROMAN 2006-The 15th IEEE International Symposium on Robot and Human Interactive Communication*. IEEE (2006). p. 558–563.

[27] Leite I Pereira A Mascarenhas S Martinho C Prada R Paiva A. The influence of empathy in human–robot relations. Int J Hum Comput Stud. (2013) 71:250–60. 10.1016/j.ijhcs.2012.09.005

[28] Schneeberger T Ehrhardt S Anglet MS Gebhard P. Would you follow my instructions if I was not human? Examining obedience towards virtual agents. In: *2019 8th International Conference on Affective Computing and Intelligent Interaction (ACII)*. IEEE (2019). p. 1–7.

[29] Vannucci F Di Cesare G Rea F Sandini G Sciutti A. A robot with style: can robotic attitudes influence human actions? In: *2018 IEEE-RAS 18th International Conference on Humanoid Robots (Humanoids)*. IEEE (2018). p. 1–6.

[30] Cameron RP Gusman D. The primary care PTSD screen (PC-PTSD): development and operating characteristics. Prim Care Psychiatry. (2003) 9:9–14. 10.1185/135525703125002360

[31] Cohn JF Kruez TS Matthews I Yang Y Nguyen MH Padilla MT, et al. Detecting depression from facial actions and vocal prosody. In: *2009 3rd International Conference on Affective Computing and Intelligent Interaction and Workshops*. IEEE (2009). p. 1–7. 10.1109/ACII.2009.5349321 PMC3296481 21278824

[32] Guohou S Lina Z Dongsong Z. What reveals about depression level? The role of multimodal features at the level of interview questions. Inf Manag. (2020) 57:103349. 10.1016/j.im.2020.103349

[33] Harrison AG Edwards MJ Parker KC. Identifying students faking ADHD: preliminary findings and strategies for detection. Arch Clin Neuropsychol. (2007) 22:577–88. 10.1016/j.acn.2007.03.008 17507198

[34] Sollman MJ Ranseen JD Berry DT. Detection of feigned ADHD in college students. Psychol Assess. (2010) 22:325. 10.1037/a0018857 20528060

[35] Costanza RS Derlega VJ Winstead BA. Positive and negative forms of social support: effects of conversational topics on coping with stress among same-sex friends. J Exp Soc Psychol. (1988) 24:182–93. 10.1016/0022-1031(88)90020-0

[36] Bolt E Ho JT Roel Lesur M Soutschek A Tobler PN Lenggenhager B. Effects of a virtual gender swap on social and temporal decision-making. Sci Rep. (2021) 11:1–15. 10.1038/s41598-021-94869-z 34321591 PMC8319130

[37] Fabri M Moore DJ Hobbs DJ. The emotional avatar: non-verbal communication between inhabitants of collaborative virtual environments. In: *International Gesture Workshop*. Springer (1999). p. 269–273.

[38] Gratch J Wang N Gerten J Fast E Duffy R. Creating rapport with virtual agents. In: *International Workshop on Intelligent Virtual Agents*. Springer (2007). p. 125–138.

[39] Chen T Peltola MJ Dunn R Pajunen SM Hietanen JK. Modulation of the eyeblink and cardiac startle reflexes by genuine eye contact. Psychophysiology. (2017) 54:1872–81. 10.1111/psyp.12975 28792611

[40] Carroll JM Russell JA. Facial expressions in Hollywood’s portrayal of emotion. J Pers Soc Psychol. (1997) 72:164. 10.1037/0022-3514.72.1.164 8636880

[41] D’Mello SK Craig SD Graesser AC. Multimethod assessment of affective experience and expression during deep learning. Int J Learn Technol. (2009) 4:165–87. 10.1504/IJLT.2009.028805

[42] Ghamen K Caplier A. Positive and negative expressions classification using the belief theory. Int J Tomogr Stat. (2011) 17:72–87. hal-00565679, version 1.

[43] Grafsgaard JF Wiggins JB Boyer KE Wiebe EN Lester JC. Automatically recognizing facial indicators of frustration: a learning-centric analysis. In: *2013 Humaine Association Conference on Affective Computing and Intelligent Interaction*. IEEE (2013). p. 159–165.

[44] Watson D Clark LA Tellegen A. Development and validation of brief measures of positive and negative affect: the PANAS scales. J Pers Soc Psychol. (1988) 54:1063. 10.1037/0022-3514.54.6.1063 3397865

[45] de Starceva-Apele A Rascevska M. Reliability and factorial validity of long and brief versions of the inventory of personality organization in a Latvian sample. Res Psychother.: Psychopathol Process Outcome. (2022) 25:159–72. 10.4081/ripppo.2022.606 PMC9422320 35796597

[46] Díaz-García A González-Robles A Mor S Mira A Quero S García-Palacios A, et al. Positive and negative affect schedule (PANAS): psychometric properties of the online Spanish version in a clinical sample with emotional disorders. BMC Psychiatry. (2020) 20:1–13. 10.1186/s12888-020-2472-1 32039720 PMC7008531

[47] Tomsone S. *Aspects of home, healthy ageing among very old Europeans: a Latvian perspective* [PhD thesis] (2009).

[48] Manea L Gilbody S McMillan D. Optimal cut-off score for diagnosing depression with the patient health questionnaire (PHQ-9): a meta-analysis. CMAJ. (2012) 184:E191–6. 10.1503/cmaj.110829 22184363 PMC3281183

[49] [Dataset] Pfizer https://www.phqscreeners.com/select-screener.

[50] Goldberg LR. A broad-bandwidth, public domain, personality inventory measuring the lower-level facets of several five-factor models. Pers Psychol Eur. (1999) 7:7–28.

[51] Strus W Cieciuch J Rowiński T. The circumplex of personality metatraits: a synthesizing model of personality based on the big five. Rev Gen Psychol. (2014) 18:273–86. 10.1037/gpr0000017

[52] Ypofanti M Zisi V Zourbanos N Mouchtouri B Tzanne P Theodorakis Y, et al. Psychometric properties of the international personality item pool big-five personality questionnaire for the Greek population. Health Psychol Res. (2015) 3:41–7. 10.4081/hpr.2015.2206 26973962 PMC4768534

[53] Zheng L Goldberg LR Zheng Y Zhao Y Tang Y Liu L. Reliability and concurrent validation of the IPIP big-five factor markers in China: consistencies in factor structure between internet-obtained heterosexual and homosexual samples. Pers Individ Dif. (2008) 45:649–54. 10.1016/j.paid.2008.07.009 20383283 PMC2851096

[54] Biderman M. Applications of bifactor models to big five data. In: *28th Annual Conference of the Society for Industrial and Organizational Psychology*; Houston, TX (2013).

[55] Kerr BA Birdnow M Wright JD Fiene S. They saw it coming: rising trends in depression, anxiety, and suicidality in creative students and potential impact of the COVID-19 crisis. Front Psychol. (2021) 485 128–44. 10.3389/fpsyg.2021.611838 PMC7956977 33732183

[56] [Dataset] Pērkona A Koļesovs K. Person$\bar{\text{i}}$bas aptaujas “starptautiskā person$\bar{\text{i}}$bas pantu kopuma (ipip-50) lielā piecinieka marķieri” adaptācija latviešu valodā (2019).

[57] Perepjolkina V Reņǵe V. Psychometric properties of the final version of a Latvian personality inventory. In: *12 th European Conference on Psychological Assessment, San Sebastian, Spain. Book of Abstracts* San Sebastian, Spain: FotocopiasZorroaga, S.L. (2013). p. 354–355.

[58] Schmitt DP Allik J McCrae RR Benet-Martínez V. The geographic distribution of big five personality traits: patterns and profiles of human self-description across 56 nations. J Cross Cult Psychol. (2007) 38:173–212. 10.1177/0022022106297299

[59] Faul F Erdfelder E Buchner A Lang A-G. Statistical power analyses using g* power 3.1: tests for correlation and regression analyses. Behav Res Methods. (2009) 41:1149–60. 10.3758/BRM.41.4.1149 19897823

[60] Hone K. Empathic agents to reduce user frustration: The effects of varying agent characteristics. Interact Comput. (2006) 18:227–45. 10.1016/j.intcom.2005.05.003

[61] Pratt JA Hauser K Ugray Z Patterson O. Looking at human–computer interface design: effects of ethnicity in computer agents. Interact Comput. (2007) 19:512–23. 10.1016/j.intcom.2007.02.003

[62] Richards D Alsharbi B Abdulrahman A. Can I help you? Preferences of young adults for the age, gender and ethnicity of a virtual support person based on individual differences including personality and psychological state. In: *Proceedings of the Australasian Computer Science Week Multiconference*. Melbourne: Association for Computing Machinery (ACM) (2020). p. 1–10.

[63] [Dataset] Unity. Toon people ver 3.1 (2021). Available from: http://jb3d.es/marketplace/

[64] [Dataset NCH Software] https://www.nchsoftware.com/voicechanger/.

[65] [Dataset] https://hugo.lv/en/About.

[66] Monster A Chan H O’Connor D. Long-term trends in human eye blink rate. Biotelem Patient Monit. (1978) 5:206–22. PMID: . 754827

[67] Tsubota K Nakamori K. Dry eyes and video display terminals. N Engl J Med. (1993) 328:584– . 10.1056/NEJM199302253280817 8426634

[68] Brugman H Russel A. Annotating multi-media/multi-modal resources with ELAN. Lisbon: European Language Resources Association (ELRA). (2004). p. 2065–2068.

[69] [Dataset] https://archive.mpi.nl/tla/elan.

[70] Baltrusaitis T Zadeh A Lim YC Morency L-P. Openface 2.0: facial behavior analysis toolkit. In: *2018 13th IEEE International Conference on Automatic Face & Gesture Recognition (FG 2018)*. IEEE (2018). p. 59–66.

[71] Baltrušaitis T Mahmoud M Robinson P. Cross-dataset learning and person-specific normalisation for automatic action unit detection. In: *2015 11th IEEE International Conference and Workshops on Automatic Face and Gesture Recognition (FG)*. Vol. 6. IEEE (2015). p. 1–6.

[72] Wood E Baltrusaitis T Zhang X Sugano Y Robinson P Bulling A. Rendering of eyes for eye-shape registration and gaze estimation. In: *Proceedings of the IEEE International Conference on Computer Vision*. Santiago: IEEE (2015). p. 3756–3764.

[73] Cohn JF Ambadar Z Ekman P. Observer-based measurement of facial expression with the facial action coding system. *The handbook of emotion elicitation and assessment*. Vol. 1. Oxford: Oxford University Press (2007). p. 203–221.

[74] Soysal ÖM Shirzad S Sekeroglu K. Facial action unit recognition using data mining integrated deep learning. In: *2017 International Conference on Computational Science and Computational Intelligence (CSCI)*. IEEE (2017). p. 437–443.

[75] Rottenberg J Gross JJ Gotlib IH. Emotion context insensitivity in major depressive disorder. J Abnorm Psychol. (2005) 114:627. 10.1037/0021-843X.114.4.627 16351385

